# Mechanistic insights into genomic structure and functions of a novel oncogene YEATS4

**DOI:** 10.3389/fcell.2023.1192139

**Published:** 2023-06-26

**Authors:** Qingqing Xian, Yiying Song, Chengzhi Gui, Yunying Zhou

**Affiliations:** ^1^ Department of Clinical Laboratory Diagnosis, Shandong University, Jinan, Shandong, China; ^2^ Department of Clinical Laboratory Diagnosis, Shandong First Medical University, Jinan, Shandong, China; ^3^ Medical Research and Laboratory Diagnostic Center, Central Hospital Affiliated to Shandong First Medical University, Jinan, Shandong, China

**Keywords:** YEATS4, oncogene, tumorigenesis, signaling pathway, regulatory mechanism

## Abstract

As a novel oncogene, the role of YEATS domain-containing protein 4 (YEATS4) in the occurrence, development, and treatment of tumors is now beginning to be appreciated. YEATS4 plays an important role in regulating DNA repair during replication. The upregulation of YEAST4 promotes DNA damage repair and prevents cell death, whereas its downregulation inhibits DNA replication and induces apoptosis. Additionally, accumulating evidence indicates that the aberrant activation of YEATS4 leads to changes in drug resistance, epithelial-mesenchymal transition and also in the migration and invasion capacity of tumor cells. Therefore, specific inhibition of the expression or activity of YEATS4 protein may be an effective strategy for inhibiting the proliferation, motility, differentiation, and/or survival of tumor cells. Taken together, YEATS4 has emerged as a potential target for multiple cancers and is an attractive protein for the development of small-molecule inhibitors. However, research on YEAST4 in tumor-related fields is limited and its biological functions, metabolism, and the regulatory mechanism of YEATS4 in numerous cancers remain undetermined. This review comprehensively and extensively summarizes the functions, structure and oncogenic roles of YEATS4 in cancer progression and aims to further contribute to the study of its underlying molecular mechanism and targeted drugs.

## 1 Background

Tumorigenesis is a complex biological process, closely related to the activation of oncogenes and the inactivation of tumor suppressor genes (Jemal et al., 2011). Chromatin modification and transcriptional regulation are crucial biological processes. Increasing number of conserved protein domains, including the YEATS domain, are involved in this process. As a member of a relatively newly discovered epigenetic reading protein family, characterized by the presence of N-terminal YEATS domain (Schulze et al., 2009), YEATS4 was first identified and isolated from glioblastoma in 1997 (Fischer et al., 1997); therefore, it is also known as the glioma amplification gene (GAS41). YEATS4 is a highly conserved nuclear protein gene, and the protein encoded by it is an important chromatin-remodeling molecule, that is, involved in epigenetic regulation. Further research has found that YEATS4 is amplified and overexpressed in various types of malignancies [such as astrocytomas, uterine fibroids, liposarcoma, breast cancer, liver cancer, pancreatic cancer, gastric cancer, non-small cell lung cancer, colorectal cancer, ovarian cancer, and other cancers (Fischer et al., 1996; Fischer et al., 1997; Debernardi et al., 2002; Barretina et al., 2010; Park et al., 2011; Schmitt et al., 2012; Pikor et al., 2013; Kim et al., 2015; Kiuchi et al., 2018; Berta et al., 2021)], resulting in malignant progression and poor prognosis of tumors.

Previous studies have revealed that YEATS4 is mainly involved in chromatin modification and transcriptional regulation (Doyon et al., 2004; Cai et al., 2005) by interacting with its target genes, MYC, KIAA1009, AF10, TACC1, NuMA, MYCN, TFIIF, PFDN1, TACC2, and AP-2 beta (Harborth et al., 2000; Debernardi et al., 2002; Lauffart et al., 2002; Ding et al., 2006; Heisel et al., 2010). In addition, YEATS4 plays an important role in the regulation of DNA repair during replication. The upregulation of YEATS4 promotes DNA damage repair and prevents cell death, whereas its downregulation inhibits DNA replication and induces apoptosis. Therefore, the development of small-molecule inhibitors targeting YEATS4 has become an attractive challenge, which can be achieved by reducing or inhibiting the expression of YEATS4 or preventing interactions between YEATS4 and other molecules. However, most current research on YEATS4 focuses on promoting cell growth and viability, while relatively few studies have been conducted on the development of small-molecule inhibitors. Notably, the biological function, precise underlying mechanism, and prognosis of YEATS4 have not yet been fully elucidated. Therefore, an intensive study needs to be conducted on YEATS4.

This review aims to provide a comprehensive overview of the function and carcinogenic effects of YEATS4 and facilitate further exploration of therapeutic targets and prognostic biomarkers for various cancers.

## 2 Functions of YEATS4

YEATS4 gene contains 103084 bases and is localized in the nucleus. It is localized on human chromosome 12q13-15, and its encoded protein consists of 227 amino acids with a molecular weight of approximately 26.7kD (Wang et al., 2018). There are four types of human YEATS domain proteins: eleven-nineteen-leukemia (ENL), ALL1-fused gene from chromosome 9 protein (AF9), YEATS2, and YEATS domain-containing protein 4 (YEATS4). The N-terminus of YEATS4 contains a highly conserved YEATS domain, a common component of the YEATS domain protein family (Harborth et al., 2000). The C-terminus is a negatively charged α-helix structure, an area of interaction between proteins, that is, species-specific (Piccinni et al., 2011). Evolutionarily, YEATS4 is highly conserved (Zimmermann et al., 2002), and as a member of the YEATS protein family, YEATS4 has high homology with AF9 and ENL. However, it also has some unique structures and functions.

YEATS4 is a structurally incomplete transcription factor that does not contain a DNA-binding domain bound to other gene promoter regions, so it cannot bind to DNA. However, it contains a DNA-activating domain that binds to and activates other transcription factors and promotes their binding to DNA, promoting the expression of target genes (Heisel et al., 2010). Yaf9 is a yeast YEATS domain protein with 80% similarity and 53% identity to YEATS4, and most similarities are found in the N-terminal region (Schulze et al., 2010). This suggests that there may also be heightened functional similarities between Yaf9 and YEATS4, which proved to be the case. In *Saccharomyces cerevisiae*, Yaf9 constitutes the histone acetyltransferase complex NuA4 and the chromatin remodeling complex SWR1 (Le Masson et al., 2003; Bianchi et al., 2004). In mammals, YEATS4 is a subunit of the SRCAP and Tip60 complexes, which correspond to NuA4 and SWR1 complexes in yeast. The involvement of Yaf9 and YEATS4 as part of a complex that can alter chromatin structure and promote gene transcription suggests that Yaf9 and YEATS4 can facilitate the localization of these complexes to the promoter regions of target genes in yeast and mammals (Zhang et al., 2004). These studies indicate that yeast may be a good choice to use as a model to study the structure, function, and mechanism of YEATS4 in mammalian cells.

Like other YEATS proteins, YEATS4 functions to reconstruct chromosome conformation and regulate transcription, and is involved in the composition of SRCAP and TIP60/TRRAP chromatin remodeling complexes (Cai et al., 2005). Spindle formation-related proteins, such as NuMA, human transformed acid coil 1 protein (TACC1),-tubulin, interact with YEATS4, which is complex and important for spindle formation. NuMA-YEATS4 imbalance may produce abnormal spindles and chromatin, which play a critical role in cell proliferation and cycle formation (Yeewa et al., 2022).

**FIGURE 1:**
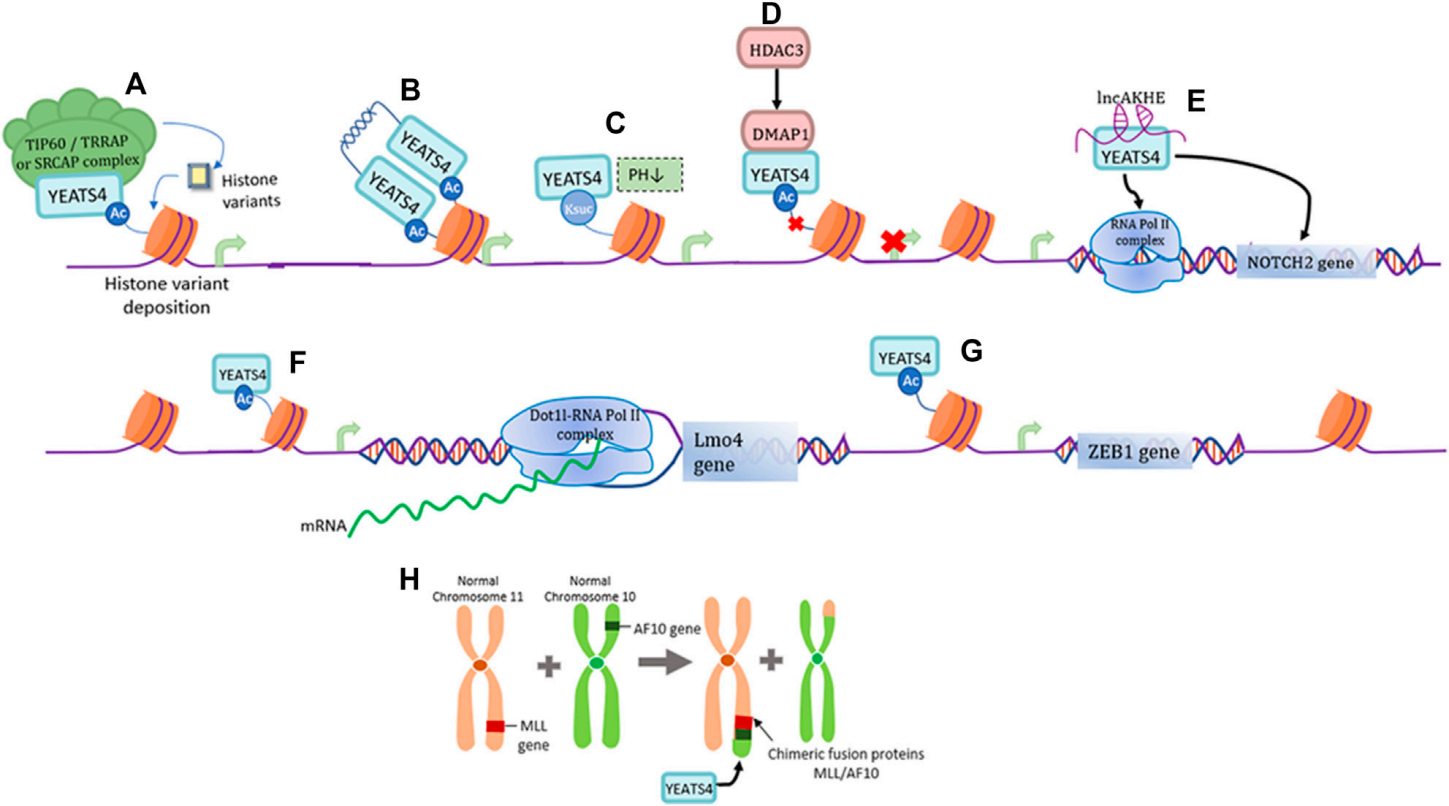
Functions of YEATS4. **(A)** YEATS4 is involved in forming part of the TIP60/TRRAP or SRCAP complex and plays an epigenetic regulatory role by recognizing histone acetylated lysines. **(B)** YEATS4 forms a dimer through the C-terminal α-helical structure, and its N-terminal highly conserved YEATS structural domain recognizes and binds diacetylated H3 with higher affinity. **(C)** Under low pH environment, YEATS4 can bind to H3K122suc and thus regulate gene expression. **(D)** HDAC3 mediates the binding of DMAP1 to YEATS4, producing inhibition of histone acetylation and gene transcription. **(E)** LncAKHE binds to YEATS4 and activates the transcription and signaling pathway of NOTCH2 gene. **(F)** YEATS4 recognition of H3K27ac drives the recruitment of Dot1l-RNA Pol II complex in the Lmo4 promoter region. **(G)** YEATS4 binds to the promoter region of ZEB1 gene and promotes ZEB1 gene expression. **(H)** MLL translocates with AF10 to produce the fusion protein MLL/AF10, where the leucine motif of AF10 is retained and the leucine zipper region of AF10 specifically interacts with the C-terminal coil structure of YEATS4.

YEATS4 is an epigenetic reader capable of recognizing acetylated lysine residues. First, YEATS4 can bind histone H3 near the promoter of the target gene through its YEATS domain, and is specific for the binding of histones H3K27ac and H3K14ac, thereby promoting the deposition of the histone variant H2A.Z and transcriptional activation of the target gene (Figure 1A) (Hsu et al., 2018). The two YEATS4 molecules can also form dimers through their α-helix structure at the C-terminus, which can bind to diacetylated H3 with higher affinity and preferentially bind to H3K18ac and H3K27ac, demonstrating a unique binding pattern of acetylated histones (Figure 1B) (Cho et al., 2018). At a lower pH, YEATS4 can also bind to succinylated lysine (Ksuc), and YEATS4 can cooperate with H3K122suc and show strong affinity (Figure 1C) (Wang et al., 2018). In addition, HDAC3 mediates transcriptional inhibition through YEATS4 and the co-inhibitor DMAP1, thereby affecting H2A acetylation and transcriptional regulation (Figure 1D) (Witt et al., 2009; Ukey et al., 2022).

lncAKHE is a long-stranded non-coding RNA (lncRNA) that binds to YEATS4 and drives enhanced recognition of the NOTCH2 promoter region by RNA Pol II, thereby promoting the transcription and translation of the NOTCH2 gene and activation of the NOTCH2 signaling pathway (Figure 1E) (Huang et al., 2018). It has also been shown that the recognition of H3K27ac by YEATS4 can promote the transcriptional expression of Lmo4 and recruit the Dot1l-RNA Pol II complex to the promoter of the Lmo4 gene, which is necessary for α4β7+CLP differentiation to ILC (Figure 1F) (Liu et al., 2019). As a member of the zinc finger protein family, ZEB1 induces epithelial-mesenchymal (EMT) transformation of tumor cells (Zhang et al., 2015). YEATS4 promotes ZEB1 expression by interacting with histone H3K27ac in the promoter region of the ZEB1 oncogene (Figure 1G) (Li et al., 2021). MLL translocates with AF10 to produce a fusion protein, MLL/AF10, in which the leucine motif of AF10 is preserved. INI1 (integrase interactor 1) is part of the important chromatin remodeling complex SWI/SNF and can interact with YEATS4 via AF10 interaction, suggesting that these three proteins may exist in the same chromatin complex and work together to influence normal gene regulatory functions (Figure 1H) (Debernardi et al., 2002).

## 3 Signaling pathways participated by YEATS4

YEATS4 plays a role in the development and progression of many cancers, and its expression is involved in tumor cell proliferation, invasion, metastasis, epithelial-mesenchymal transition transformation, and treatment resistance. Previous studies have shown that, as a transcriptional activator, YEATS4 is mainly involved in P53 pathway, NOTCH pathway, and-catenin pathways.

### 3.1 P53 pathway

**FIGURE 2:**
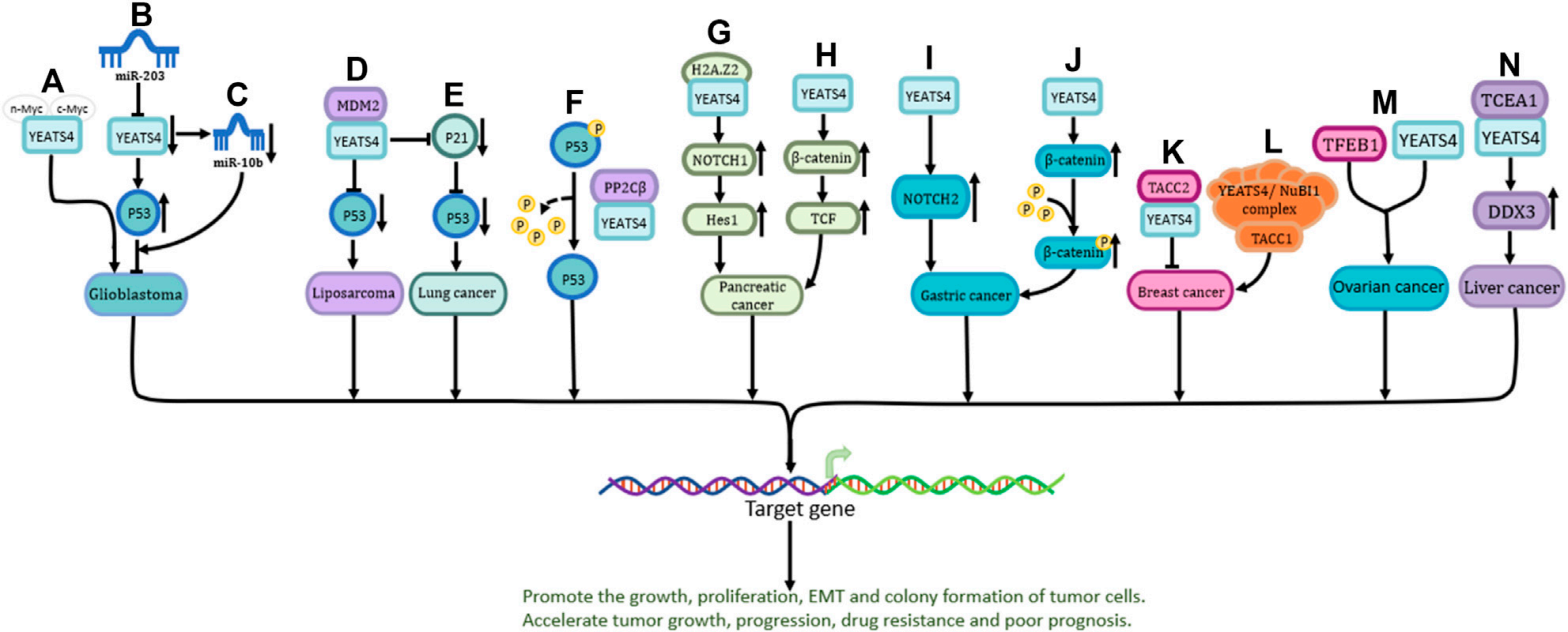
The role and the pathways of YEATS4 involved in tumors. **(A)** n-Myc and c-Myc play a role in glioblastoma by binding to the C-terminal region of YEATS4. **(B)** miR-203 indirectly enhances P53 activity through suppressing YEATS4 expression and exerts inhibitory effects. **(C)** Reduced YEATS4 expression causes downregulation of miR-10b expression and inhibits the progression of glioblastoma. **(D)** MDM2 cooperates with YEATS4 to attenuate the activity of P53 protein and promote the process of liposarcoma. **(E)** YEATS4 indirectly down-regulates P53 through down-regulation of P21 protein in lung cancer. **(F)** The GAS41-PP2Cβ complex specifically dephosphorylates p53 at serine 366. **(G)** YEATS4 binds H2A.Z2 and promotes activation of NOTCH1, leading to upregulation of downstream Hes1 expression. **(H)** Overexpression of YEATS4 activates the Wnt/β-catenin signaling pathway and promotes the proliferation of pancreatic cancer cells**. (I)** YEATS4 upregulates NOTCH2 expression and activates NOTCH2 signaling pathway in gastric cancer. **(J)** Transforming acidic coil-containing protein 2 (TACC2) forms a complex with YEATS4 to affect the growth and spread of pancreatic cancer cells. **(K)** TACC2 forms a complex with YEATS4 to affect the growth and spread of breast cancer cells. **(L)** TACC1 acts together with the YEATS4/NuBI1 complex, in which YEATS4 acts as a bridge protein to promote the expression of downstream target genes. **(M)** Combined action of TFEB1 and YEATS4 in ovarian cancer cells. **(N)** YEATS4 binds and activates transcription elongation factor A1 (TCEA1), which in turn promotes the upregulation of DDX3 (DEAD box protein 3) expression and plays an important role in hepatocellular carcinoma.

P53 is one of the most important tumor suppressor genes (Vodicka et al., 2021). YEATS4 promotes cancer development by inhibiting the activity of P53 (Llanos et al., 2006). The C-terminus of YEATS4 is required for its interaction with MYC proteins (including n-Myc and c-Myc) (Figure 2A) (Piccinni et al., 2011). In addition, YEATS4 (suppressed by miR-203) inhibits the development of glioblastoma by directly enhancing the activity of the p53 protein (Figure 2B) or indirectly downregulating the expression of miR-10b (Figure 2C) (Pal et al., 2016).

MDM2, a key suppressor of P53, is upregulated in various tumors (Oliner et al., 2016). Amplified MDM2 binds to YEATS4, inhibits P53 protein activity, and accelerates the malignant progression of liposarcoma (Figure 2D) (Italiano et al., 2008). In non-small cell lung cancer, YEATS4 has been shown to act synergistically with MDM2 to inhibit P53 activity by inhibiting P21 (Figure 2E) (Pikor et al., 2013). P53 is degraded by the proteasome, and the complex formed by YEATS4 and PP2Cβ can dephosphorylate the serine residue at 366-position of P53, resulting in increased P53 instability (Figure 2F) (Park et al., 2011). YEATS4 inhibits the P53 pathway and promotes cell proliferation during cell division, which is of great significance in the occurrence of a variety of cancers (Park and Roeder, 2006).

### 3.2 NOTCH pathway

The Notch signaling pathway is highly conserved, plays a vital role in many life processes, and is closely related to cell growth, development, survival, and drug resistance (Bray, 2016). YEATS4 can bind to H2A.Z2, which in turn drives the activation of NOTCH1, upregulates the expression of NOTCH1 and its downstream mediator Hes1, and mediates the occurrence and drug resistance of pancreatic cancer (Figure 2G) (Han et al., 2022). In addition, overexpression of YEATS4 in gastric cancer cells activates NOTCH2, promoting cancer cell proliferation and poor prognosis (Figure 2I) (Kiuchi et al., 2018).

### 3.3 Wnt/β-catenin pathway

The Wnt/β-catenin pathway is the most characteristic Wnt signaling pathway and plays a crucial role in human embryonic development and homeostatic regulation (Sharma and Chopra, 1976; Liu et al., 2022; Hashemi et al., 2023). Overexpression of YEATS4 activates the-catenin/TCF signaling pathway and promotes the proliferation, invasion, and migration of pancreatic cancer cells (Figure 2H) (Jixiang, Shengchun, Jianguo, Zhengfa, Xin, Xuqing, et al.). In gastric cancer, upregulated YEATS4 directly acts on β-catenin to activate its transcription, thereby promoting the protein expression of β-catenin and inducing its phosphorylation (Figure 2J) (Ji et al., 2017). The specific mechanism of action of YEATS4 in the-catenin pathway however requires further investigation.

### 3.4 YEATS4 and other pathways

In breast cancer, YEATS4 recognizes H3K27ac in the ZEB1 promoter and promotes EMT in breast cancer cells (Figure 1G) (Zhang et al., 2015). Additionally, TACC1 and YEATS4 regulate the expression of downstream target genes and accelerate breast cancer malignancy (Figure 2L) (Lauffart et al., 2002). YEATS4 and TFEB1 have been shown to be upstream transcription factors in ovarian cancer (Figure 2M) (Kim et al., 2015). Moreover, YEATS4 can bind and activate transcriptional elongation factor A1 (TCEA1), which then promotes the upregulation of DDX3, a DEAD-box family RNA helicase with multiple cellular functions that plays an important role in liver cancer (Figure 2N) (You et al., 2018).

## 4 YEATS4 and human cancer

Numerous studies have shown that YEATS4 is amplified during cancer progression (Vogelstein et al., 2013). In contrast, the knockdown of YEATS4 was followed by the suppression of all typical features of tumor cells, suggesting that YEATS4 may be a potential therapeutic target and a prognostic biomarker for cancer. However, according to the available experimental results, the molecular mechanisms of YEATS4 in different types of tumor cells are not the same (Table 1).

**TABLE 1:** YEATS4 and its partners and their functions in cancer.

Tumor type	Targets/Regulators and signaling pathways	Function	References
Glioblastoma	n-Myc, c-Myc, miR-203, miR-10b, NuMA, TACC1, γ-tubulin	↑↑YEATS4: ↑ n-Myc, c-Myc, △miR-203: ↑YEATS4, miR-10b, ↓apoptosis, ↑migration and invasion of cell, ↑↑YEATS4: formation of multi-polar spindles	Pal et al. (2016), Kang et al. (2023)
Uterine leiomyoma	H2A	mutation in YEATS4: insufficient deposition of H2A.Z, increased prevalence	Berta et al. (2021)
Liposarcoma		↑↑YEATS4: associated to the formation of giant rings or giantrod-labeled chromosomes	Persson et al. (2008), Pei et al. (2021), Mashima et al. (2021)
Breast cancer	ZEB1, TACC1, TACC2	↑↑YEATS4: ↑EMT, migration, invasion, and transfer, ↓YEATS4: ↓growth, migration, progression of the cancer	Lauffart et al. (2002), Lauffart et al. (2003), Li et al. (2021)
Liver cancer	TCEA1/DDX3, lncAKHE, NOTCH signaling pathway	↑↑ YEATS4: ↑cell proliferation, migration, invasion and colony formation, ↑the occurrence and development of cancer	Huang et al. (2018), You et al. (2018), Tao et al. (2022)
Pancreatic cancer	β-catenin/TCF signaling pathway, H3, NOTCH signaling pathway	↑↑YEATS4: ↑malignant proliferation, invasion and migration of cancer cells, ↑cell stemness and GEM resistance	Valenzuela et al. (2014), Han et al. (2022), Jixiang et al. (2017)
Gastric cancer	Wnt/β-Catenin signaling pathway	↑↑YEATS4: ↑cell viability, colony formation, ↓apoptosis, ↑malignant progression and poor prognosis	Ji et al. (2017), Kiuchi et al. (2018), Rivera-Yañez et al. (2023)
Lung cancer	H3K27, H3K14, P53 signaling pathway	↑↑YEATS4: ↑ growth of tumors and resistance to cisplatin, ↓aging and apoptosis of cells	Pikor et al. (2013), Hsu et al. (2018)
Colorectal cancer	miR-218	↓↓ YEATS4: ↑apoptosis, ↓drug resistance to L-OHP, cytoprotective autophagy, progression of cancer	Tao et al. (2015), Fu et al. (2016), Luque et al. (2022)
Leukaemia	AF10, INI1	associated with the development of leukemia	Debernardi et al. (2002), Caudell and Aplan (2008), Hagen et al. (2014), Marschalek (2015)
Ovarian cancer	TFEB1	↑↑ YEATS4: ↑drug resistance, ↓treatment efficiency	Kim et al. (2015), Fidahussain et al. (2022)

△, knock-down or deletion; ↑, amplified or overexpressed or enhanced; ↓, knock down or cut or weaken.

### 4.1 Glioblastoma

In glioblastoma, YEATS4 can cooperate with n-Myc and c-Myc through its C-terminus (Piccinni et al., 2011; Kang et al., 2023) and can also be repressed by miR-203, which in turn mediates a decrease in miR-10b expression. Downregulation of YEATS4 diminishes the inhibitory effect on the P21/P53 pathway, impairing apoptosis, migration, and invasion of glioblastoma cells (Pal et al., 2016).

### 4.2 Uterine leiomyoma

Kaasinen et al. found that the deposition defect of H2A.Z is one of the factors that cause uterine fibroids, and the complex mutation of SRCAP members can cause insufficient deposition of H2A.Z, among these, YEATS4, and ZNHIT1 mutations are the most strongly correlated, resulting in an increased probability of uterine fibroids (Berta et al., 2021). Although this study links complex genetic mutations in SRCAP to the occurrence of uterine fibroids, further research is required to understand the molecular mechanisms involved.

### 4.3 Liposarcoma

As mentioned above, in liposarcomas, MDM2 and YEATS4 amplifications appear to be particularly important and can form characteristic giant ring or giant rod marker chromosomes (Persson et al., 2008; Pei et al., 2021), suggesting that YEATS4 can be used as a diagnostic and therapeutic target for liposarcoma (Mashima, Sawada, Nakamura).

### 4.4 Breast cancer

In breast cancer, YEATS4 recognizes histone H3K27ac in the promoter region of ZEB1, thereby promoting ZEB1 expression and accelerating tumor progression (Li et al., 2021). Dysregulation of the human transforming acidic coiled-coil (TACC) protein is associated with the development and progression of breast cancer, where both TACC1 and TACC2 bind to YEATS4 to form complexes that affect the growth and proliferation of breast cancer cells (Lauffart et al., 2002; Lauffart et al., 2003). TACC1 interacts with the YEATS4/NuBI1 complex, regulates the expression of downstream genes, and promotes tumorigenesis (Lauffart et al., 2002). In contrast, TACC2 binds to and inhibits YEATS4 in breast cancer cell lines, attenuating tumor growth and migration (Lauffart et al., 2003).

### 4.5 Liver cancer

TCEA1, an isoform of transcription factor SOX, is overexpressed in hepatocellular carcinoma. DDX3 (DEAD box protein 3) is involved in post-transcriptional processes and is aberrantly expressed in various tumors. Li et al. found that YEATS4 expression is upregulated in hepatocellular carcinoma tissues and binds to a specific site in the TCEA1 promoter (Tao et al., 2022), inducing the upregulation of TCEA1 gene expression, which in turn increases DDX3 expression and accelerates the proliferation and migration of hepatocellular carcinoma cells (You et al., 2018). Moreover, lncAKHE binds to YEATS4 and promotes the binding of the NOTCH2 gene promoter region to RNA Pol II, activating the NOTCH2 signaling pathway (Huang et al., 2018).

### 4.6 Pancreatic cancer

Pancreatic cancer has an insidious onset and extremely poor prognosis (Valenzuela et al., 2014). It was found that upregulation of YEATS4 in pancreatic cancer promotes the growth, proliferation, and migration of pancreatic cancer cells, and impairs the action of oncogenic Ras after knockdown of the YEATS4 gene, which inhibits the proliferation and transformation of pancreatic cancer cells. YEATS4 acts on-catenin/TCF signaling pathway (Jixiang, Shengchun, Jianguo, Zhengfa, Xin, Xuqing, et al.) and the NOTCH pathway, promoting the expression of downstream genes and resulting in poor prognosis in pancreatic cancer (Han et al., 2022).

### 4.7 Gastric cancer

Upregulated YEATS4 enhances Wnt/β-Catenin signaling and increases the proliferative activity, invasion, and metastatic ability of gastric cancer cells (Ji et al., 2017; Rivera-Yañez et al., 2023). YEATS4 is also associated with tumor size, depth, distant metastasis, and poor prognosis (Kiuchi et al., 2018), suggesting that YEATS4 could be a target for developing oncological drugs and improving the prognosis of gastric cancer.

### 4.8 Lung cancer

In non-small cell lung cancer (NSCLC), overexpression of YEATS4 decrease P53 inhibited cancer cell senescence and apoptosis and enhances the resistance of tumor cells to chemotherapeutic agents such as cisplatin (Pikor et al., 2013). As a member of the YEATS protein family, YEATS4 forms a chromatin-remodeling complex with SRCAP or P400, and the N-terminal of YEATS structural domain specifically recognizes and binds to lysine 27 and lysine 14 of acetylated histone H3, promoting the deposition of the histone variant H2A.Z, loosening the chromatin structure, making it easier to bind to target genes, promoting their transcription and expression, and promoting the progression of NSCLC (Hsu et al., 2018).

### 4.9 Colorectal cancer

Deng et al. found significantly higher levels of YEATS4 in colorectal cancer tissues than in normal para-cancerous tissues; however, this did not affect the overall survival of patients. When YEATS4 is knocked down in colorectal cancer cells, the cells are arrested in the G0/G1 phase and cell growth is inhibited (Tao et al., 2015; Luque et al., 2022). Subsequent studies have indicated that miR-218 can negatively regulate YEATS4 expression, thereby attenuating cellular resistance to L-OHP, impairing cellular autophagy protection, and inhibiting colorectal cancer progression (Fu et al., 2016).

### 4.10 Leukaemia

MLL and CALM are believed to translocate with the AF10 gene to produce fusion proteins MLL/AF10 and CALM-AF10, respectively, which are closely associated with the occurrence of acute leukemia (Caudell and Aplan, 2008; Marschalek, 2015). The leucine zipper sequence of AF10 is partially retained in the fusion protein and interacts with YEATS4, which then binds INI1 and cooperate with the SWI/SNF complex. However, their biological effects require further investigation (Debernardi et al., 2002; Hagen et al., 2014).

### 4.11 Ovarian cancer

YEATS4 and TFEB1 have been shown to be upstream transcription factors (TFs) that regulate drug resistance in ovarian cancer (Fidahussain et al., 2022), which is an important factor contributing to poor treatment outcomes in ovarian cancer (Kim et al., 2015).

In summary, YEATS4 overexpression is involved in the development of many cancers, including glioblastoma. However, the study of its molecular mechanism is relatively incomplete, and understanding the research progress of YEATS4 is beneficial for us to better carry out future experimental work.

## 5 Perspective

As a newly emerging oncogene, YEATS4 plays a vital role in promoting the occurrence of various tumors. The molecular mechanisms of YEATS4 in various cancers are also different, and similarities in molecular mechanisms or interactions in other cancers require further validation. Despite chemotherapy, radiotherapy, and comprehensive treatment, cancer remains the leading cause of death worldwide, and many cancers are difficult to detect early and have a poor prognosis. If we can intervene at the level of genes related to the onset and progression of tumors, it will open up new avenues for the treatment of tumors.

Based on the molecular mechanism of YEATS4’s role in cancer, the development of drugs targeting YEATS4 to inhibit its activity and function may exert an effect on cancer treatment. This can be done primarily by screening for compounds capable of acetylation with histone lysine at specific sites, thereby neutralizing the transcriptional activation of target genes (Londregan et al., 2022). The channel-like structure formed by the acetolysine-binding site of the YEATS domain, although partially exposed to solvents, does not form deep pockets, which presents a significant obstacle to the development of small-molecule conjugates for YEATS4 (Linhares et al., 2020). Dymytrii et al. designed a dimer inhibitor based on fragment screening to bind to dimerized YEATS4, block the acetyl-lysine binding channel of the YEATS domain, and impair the recognition and binding of acetylated histone H3 by YEATS4, thereby inhibiting the proliferation of non-small cell lung cancer cells (Listunov et al., 2021). However, the low activity of such inhibitors is not sufficient to achieve a potency comparable to that of bromodomain inhibitors, indicating a significant challenge in developing molecular drugs targeting YEATS4.

Nevertheless, more effective small-molecule inhibitors targeting YEATS4 should be developed to provide opportunities for drug design for cancer therapy. Further studies are needed to verify the potential interaction mechanisms between YEATS4 and other proteins in other cancers.
