# Supplementary material for: Mechanistic insights into genomic structure and functions of a novel oncogene YEATS4
Source: Front Cell Dev Biol. 2023 Jun 26;11:1192139. doi: 10.3389/fcell.2023.1192139 (PMC10332269; doi:10.3389/fcell.2023.1192139)
Supplement: Supplementary file 1 [file Table1.DOC]

**Table 1.** **YEATS4 and its partners and their functions in cancer** (△: knock-down or deletion, ↑: amplified or overexpressed or enhanced, ↓: knock down or cut or weaken)

| **Tumor Type** | **Targets/Regulators and Signaling Pathways** | **Function** | **Reference** |
| --- | --- | --- | --- |
| Glioblastoma | n-Myc, c-Myc, miR-203, miR-10b, NuMA, TACC1, γ-tubulin | ↑↑YEATS4: ↑ n-Myc, c-Myc  △miR-203: ↑YEATS4, miR-10b, ↓apoptosis, ↑migration and invasion of cell, ↑↑YEATS4: formation of multi-polar spindles | (37, 50) |
| Uterine leiomyoma | H2A | mutation in YEATS4: insufficient deposition of H2A.Z, increased prevalence | (4) |
| Liposarcoma |  | ↑↑YEATS4: associated to the formation of giant rings or giantrod-labeled chromosomes | (51-53) |
| Breast cancer | ZEB1, TACC1, TACC2 | ↑↑YEATS4: ↑EMT, migration, invasion, and transfer, ↓YEATS4: ↓growth, migration, progression of the cancer | (17, 34, 54) |
| Liver cancer | TCEA1/ DDX3, lncAKHE, NOTCH signaling pathway | ↑↑ YEATS4: ↑cell proliferation, migration, invasion and colony formation, ↑the occurrence and development of cancer | (31, 48, 55) |
| Pancreatic cancer | β-catenin/TCF signaling pathway, H3, NOTCH signaling pathway | ↑↑YEATS4: ↑malignant proliferation, invasion and migration of cancer cells, ↑cell stemness and GEM resistance | (42, 46, 56) |
| Gastric cancer | Wnt/β-Catenin signaling pathway | ↑↑YEATS4: ↑cell viability, colony formation, ↓apoptosis, ↑malignant progression and poor prognosis | (7, 47, 57) |
| Lung cancer | H3K27, H3K14, P53 signaling pathway | ↑↑YEATS4: ↑ growth of tumors and resistance to cisplatin, ↓aging and apoptosis of cells | (8, 27) |
| Colorectal cancer | miR-218 | ↓↓ YEATS4: ↑apoptosis, ↓drug resistance to L-OHP, cytoprotective autophagy, progression of cancer | (58-60) |
| Leukaemia | AF10, INI1 | associated with the development of leukemia | (9, 61-63) |
| Ovarian cancer | TFEB1 | ↑↑ YEATS4: ↑drug resistance, ↓treatment efficiency | (6, 64) |
